# Detection and localisation of hesitant steps in people with Alzheimer's disease navigating routes of varying complexity

**DOI:** 10.1049/htl.2018.5034

**Published:** 2019-04-24

**Authors:** Ian McCarthy, Tatsuto Suzuki, Catherine Holloway, Teresa Poole, Chris Frost, Amelia Carton, Nick Tyler, Sebastian Crutch, Keir Yong

**Affiliations:** 1 Pedestrian Accessibility and Movement Environment Laboratory, Department of Civil Environmental and Geomatic Engineering, University College London, London N19 5UN, UK; 2 UCL Interaction Centre, Department of Computer Science, University College London, London, UK; 3 Department of Medical Statistics, Faculty of Epidemiology and Public Health, London School of Hygiene & Tropical Medicine, London, UK; 4 Dementia Research Centre, Institute of Neurology, University College London, London, UK

**Keywords:** statistical analysis, diseases, gait analysis, biomedical measurement, patient monitoring, walking paths, step hesitancy, route complexity, Alzheimer's disease navigating routes, abnormal gait parameters, gait irregularities, adaptive locomotor responses, spatial navigation, corridors, dog-leg, counterbalanced repeated-measures design, posterior cortical atrophy, shoe-mounted inertial measurement units, step time data, position data, Alzheimer's disease, gait characteristics, IMU acceleration, statistical analysi

## Abstract

People with Alzheimer's disease (AD) have characteristic problems navigating everyday environments. While patients may exhibit abnormal gait parameters, adaptive gait irregularities when navigating environments are little explored or understood. The aim of this study was to assess adaptive locomotor responses of AD subjects in a complex environment requiring spatial navigation. A controlled environment of three corridors was set up: straight (I), U-shaped (U) and dog-leg (S). Participants were asked to walk along corridors as part of a counterbalanced repeated-measures design. Three groups were studied: 11 people with posterior cortical atrophy (PCA), 10 with typical Alzheimer's disease (tAD) and 13 controls. Spatio-temporal gait parameters and position within the corridors were monitored with shoe-mounted inertial measurement units (IMUs). Hesitant steps were identified from statistical analysis of the distribution of step time data. Walking paths were generated from position data calculated by double integration of IMU acceleration. People with PCA and tAD had similar gait characteristics, having shorter steps and longer step times than controls. Hesitant steps tended to be clustered within certain regions of the walking paths. IMUs enabled identification of key gait characteristics in this clinical population (step time, length and step hesitancy) and environmental conditions (route complexity) modifying their expression.

## 1 Introduction

Early and common symptoms of Alzheimer's disease (AD) are problems in spatial navigation, greatly undermining autonomy. While such problems are often associated with deficits in memory and planning, the role of dementia-related visual processing impairments in limiting navigation, particularly in familiar environments, is often under-recognised [1]. In typical Alzheimer's disease (tAD), initial pathological changes in medial temporal regions particularly associated with episodic and spatial memory ultimately progress to posterior cortical regions, including those supporting visuospatial processing [2]. A neurodegenerative syndrome offering important insights into dementia-related visual impairment is posterior cortical atrophy (PCA) [3]. While PCA is most commonly caused by AD, in contrast to tAD, PCA patients demonstrate relatively well-preserved memory, particularly in early disease stages, but exhibit a range of complex visual deficits and environmental disorientation [4–7].

Gait function has also been shown to be impaired in dementia. Indeed, it has even been suggested that gait could be used as a biomarker of cognitive impairment and dementia syndromes [8]. Changes in step length, step time and walking speed have been observed in people with both cognitive impairment and dementia [9], and gait changes appear to be related to the stage of the disease [10–12]. Although a basic gait laboratory setting has been used to assess gait in tAD while also performing a cognitive task [13, 14], this does not replicate the daily navigational challenges of people with visual and/or cognitive problems. Perceptual factors (e.g. lighting, clutter) may have a disproportionate effect on functional performance in tAD, but situations that replicate these conditions are difficult to set up in a standard gait laboratory. Creating environments with walls and confined spaces makes it very difficult to use standard opto-electronic motion capture systems, which require a line-of-sight view from the motion detection camera to the subject.

Sensors attached directly to the participants would, in principle, enable assessment of spatio-temporal gait characteristics under any environment. Inertial measurement units (IMUs) comprise systems of orthogonal tri-axial accelerometers, gyroscopes and sometimes magnetometers, and can provide a portable means of measuring movement. IMUs have been used in studies of gait in healthy ageing [15, 16]. Their use in the assessment of gait in Parkinson's disease and dementia has recently been described [17]. IMUs can also provide an infrastructureless means of tracking the position of first responders within buildings in emergency situations [18], and for tracking people outdoors when GPS signals are not available [19]. IMUs therefore have the potential of being able to measure simultaneously both spatio-temporal characteristics of participants’ gait and their location.

This study is part of a more general investigation of gait and spatial navigation in people with dementia in a controlled real-world environment. It is proposed that people with PCA and tAD would exhibit inefficient adaptation of gait in response to local environments (corridor turns). In this present study, we investigate the feasibility of using IMUs to detect and localise hesitant behaviour.

## 2 Methods

### 2.1 Testing environment

**Fig. 1:**
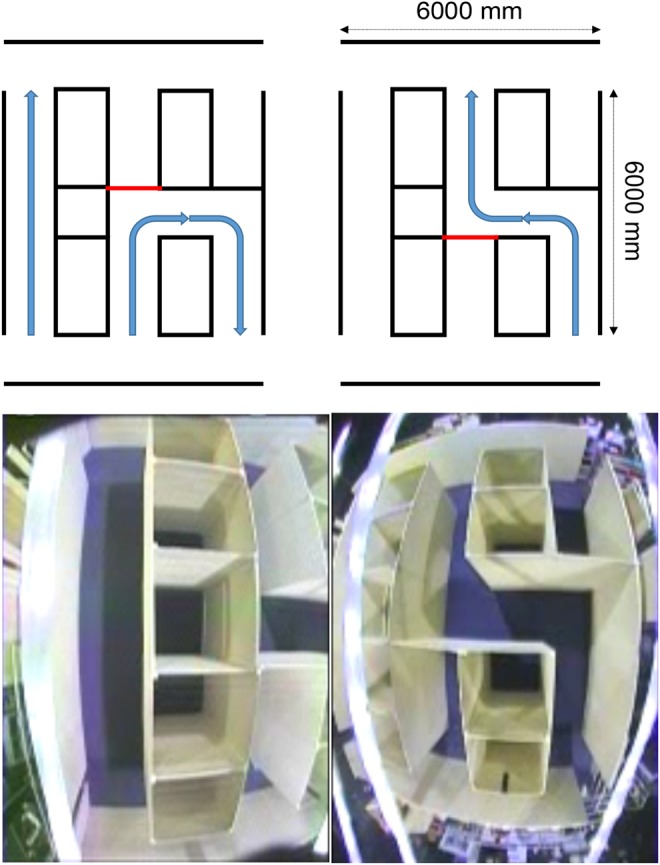
Schematic illustration of the corridor arrangement (top), and fish-eye camera view of PAMELA platform showing the boxes and panels used to construct the corridors (bottom). The arrangement allowed U- and S-shaped corridors to be created by the movement of one panel, marked in red in the schematic view

The main platform of PAMELA consisted of 54 (9 × 6) configurable 1200 mm × 1200 mm modules. Wood boards of 55 mm in thickness were put on top of the concrete modules and then covered with dark-blue domestic carpet. The entire area of floor was gap-free and levelled. The platform was configured to provide three corridors with 2.1 m high walls using free-standing wooden panels (2100 mm height, 1200 mm width, 42 mm thickness) as walls. Walls were of good contrast to the floor carpet, consistent with residential design recommendations for individuals with sight loss and dementia [20]. The three corridors comprised: a 6 m straight (I) corridor, a 8.4 m U-shaped corridor with two 90° turns in the same direction and a 8.4 m ‘dog-leg’ (S) corridor with two 90° turns in opposite directions. These are illustrated schematically in Fig. 1, along with an overhead image of the set-up. The average ground illuminance was set at 190 lux, which is a typical indoor lighting level for homes.

### 2.2 Participants and procedures

Two groups of dementia patients were studied: 11 with PCA [mean age: 64.6 ± 5.6 years; male/female: 5/6; height (cm): 168.9 ± 6.5; mini mental state examination (MMSE) score: 18.4 ± 5.9], and 10 with tAD (mean age: 66.2 ± 5.0 years; male/female: 4/6; height: 167.9 ± 11.8; MMSE: 18.6 ± 4.9). These were compared with a control group (*n* = 13, mean age: 64.2 ± 4.1 years; male/female: 7/6; height: 171.2 ± 12.9); groups were well-matched on age, height and gender. PCA and tAD patients fulfilled consensus criteria for PCA and NIA-AA criteria for tAD, respectively. Exclusion criteria were a history of other neurological or major psychiatric diseases. Patients did not fulfil clinical criteria for dementia with Lewy bodies, Parkinson's disease, corticobasal degeneration or prion disease or exhibit associated clinical features (e.g. early visual hallucinations, parkinsonism, ataxia). Each participant had IMUs attached to each heel, as described above. Participants were asked to ‘keep walking until you reach the end of the corridor’. The start of each trial was verbally signalled by the experimenter (‘Start’) preceded by counting down from three. Trials ended when participants crossed the finishing line of the route (0.6 m from the end of each corridor). Participants walked down each of three route shapes in both directions (outward and return), resulting in a total of six trials for each participant. Data for the outward direction in one trial for one participant were rejected for technical reasons. Order of route and direction conditions were counterbalanced between participants to control for order effects. Ethical approval for the study was provided by the National Research Ethics Service London-Queen Square Ethics Committee and written informed consent was obtained from all participants.

### 2.3 Assessment of movement with IMUs

**Fig. 2:**
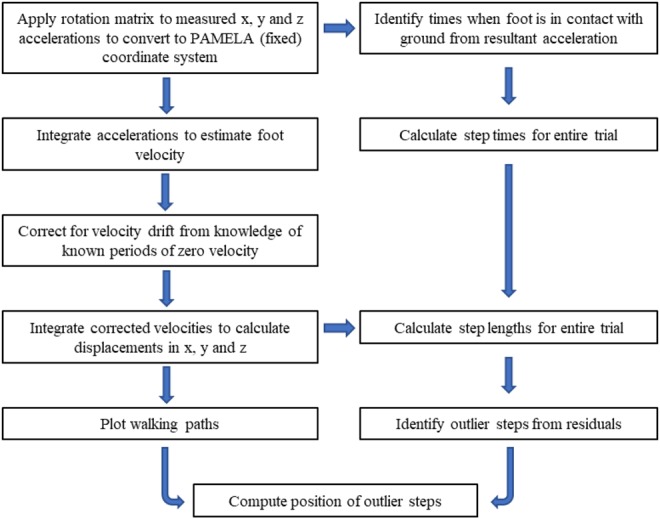
Flow diagram outlining computational stages in detecting and locating hesitant steps, starting from the box at the top left

The measurement technique has been outlined previously [21]. IMUs (Xsens, The Netherlands) were attached to the heel of each foot. Local accelerations were converted to the laboratory frame with a 3D rotation matrix, using orthogonal acceleration data combined with roll, pitch and yaw values provided by the IMU software. Stance phases for each foot were identified from resultant acceleration values below 1 m/s^2^. Initial calculations of foot velocity were performed by integration of the orthogonal acceleration data. Sensor drift was then corrected using zero velocity updates applied to identified stance phases, and the corrected velocity was integrated to provide displacement in three orthogonal directions [18, 19]. A 2D rotation was then applied to the walking paths to align them with the laboratory orientation. Step length and step time were computed for each step during these walking tasks, together with position on the platform. Outlier steps were identified using a statistical method outlined below. For a summary of stages in outlier identification and localisation, see Fig. 2.

### 2.4 Statistical analyses

All statistical analyses were carried out using Stata V.14. Descriptive statistics for step times and step lengths were calculated by group for each of the corridor configurations (I-, U- and S-shaped), expressed as the mean and standard deviation of the within person means (ignoring journey direction).

To identify and remove outlying *long* step times (i.e. hesitant steps), an iterative procedure was used as follows. For each of the ‘group by route’ combinations, a three-level linear mixed model that included random person and (within person) journey effects was fitted in order to allow for correlations between repeated measurements from the same subject, and between measurements for the same journey. Outliers with long step times were defined as observations with a standardised residual greater than 3; these outliers were dropped and the model refitted and outlier removal repeated until no further outliers were identified. A similar process was used to identify outlier *short* step lengths (i.e. steps covering a short distance), defined as those with a standardised residual less than −3.

Having removed outliers, subsequent comparisons of person-specific means by group and by route shape were performed as follows. First, for a given route shape (I-, U- or S), comparisons between participant groups (PCA, tAD, controls) were made: (i) for mean step times using a generalised least squares linear regression that allowed for the apparent different variability of groups; and (ii) for mean step lengths using an ordinary least squares linear regression. In each case, pairwise comparisons were made only if a global test for a difference was statistically significant. Second, for a given participant group (PCA, tAD, controls), comparison across route shapes (I-, U- or S-corridor) were made: (iii) for mean step times and (iv) mean step lengths, using Wilcoxon matched-pairs signed-ranks tests. For all study analyses, statistical significance was set at *p* < 0.05 (two-tailed test). There was no adjustment for multiple testing.

## 3 Results

### 3.1 Spatio-temporal gait parameters

**Fig. 3:**
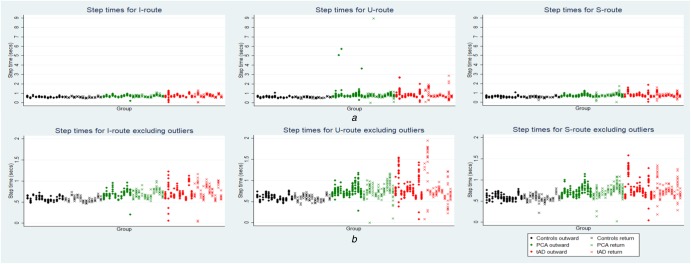
Scatter plots of step time for corridor conditions by participant group. Plots show individual step times per participant per trial *a* Including outliers *b* Excluding outliers (step times >3 standardised residuals mean for each participant)

Descriptive statistics of the spatio-temporal parameters of gait, excluding outliers as defined in terms of standardised residuals described in ‘Statistical analysis’ above, are shown in Table 1. Within the trials, there was some marked variability of step time and step length, particularly for the U- and S-corridors. This is illustrated in Fig. 3 for step time, in which individual step times for each participant are plotted; there are clearly a small number of outlier values. A total of 37 out of 2395 step times (1.5%) were considered outliers, 12/626 (1.9%), 17/863 (2.0%) and 8/906 (0.9%) for the straight, U- and S-shaped corridors, respectively (see Table 2 for group level outlier numbers for step times and lengths). Outliers were not included in the subsequent statistical analysis of group and route comparisons.

**Table 1:** Mean (standard deviation) of the observed person-specific mean step times (top) and step lengths (bottom) for the three participant groups walking under each of the three route conditions, excluding outlier values

	Step time, s			
	I-ROUTE	U-ROUTE	S-ROUTE	Step time comparison
controls	0.576 (0.052)	0.588 (0.053)	0.585 (0.060)	*U* versus *I*: *p* = 0.064
				*S* versus *I*: *p* = 0.184
				*S* versus *U*: *p* = 0.780
PCA	0.691* (0.075)	0.743* (0.090)	0.724* (0.076)	*U* versus *I*: *p* = 0.016
				*S* versus *I*: *p* = 0.062
				*S* versus *U*: *p* = 0.182
tAD	0.730* (0.122)	0.803* (0.197)	0.759* (0.153)	*U* versus *I*: *p* = 0.241
				*S* versus *I*: *p* = 0.647
				*S* versus *U*: *p* = 0.386

**Table:** 

	Step length, m			
	I-ROUTE	U-ROUTE	S-ROUTE	Step length comparison
controls	0.738 (0.128)	0.689 (0.132)	0.688 (0.127)	*U* versus *I*: *p* = 0.003
				*S* versus *I*: *p* = 0.005
				*S* versus *U*: *p* = 0.861
PCA	0.503* (0.101)	0.405* (0.122)	0.430* (0.111)	*U* versus *I*: *p* = 0.003
				*S* versus *I*: *p* = 0.003
				*S* versus *U*: *p* = 0.131
tAD	0.548* (0.073)	0.476* (0.143)	0.482* (0.104)	*U* versus *I*: *p* = 0.013
				*S* versus *I*: *p* = 0.013
				*S* versus *U*: *p* = 0.879

For each participant group, *p*-values are for Wilcoxon matched-pairs signed-rank tests comparing route shapes. Asterisks denote group differences (versus controls: *p ≤ 0.001).

**Table 2:** Number of outliers/total observations (%) by participant group and route shape for step times (top) and step lengths (bottom)

	Step time		
	I-ROUTE	U-ROUTE	S-ROUTE
control	8/191 (4.2%)	2/237 (0.8%)	1/258 (0.4%)
PCA	2/238 (0.8%)	9/354 (2.5%)	5/368 (1.4%)
tAD	2/197 (1.0%)	6/272 (2.2%)	2/280 (0.7%)

**Table:** 

	Step length		
	I-ROUTE	U-ROUTE	S-ROUTE
control	4/191 (2.1%)	0/237 (0%)	1/258 (0.4%)
PCA	5/238 (2.1%)	0/354 (0%)	0/368 (0%)
tAD	7/197 (3.6%)	0/272 (0%)	0/280 (0%)

Comparison between groups for each individual route shape showed that mean step time was significantly longer and mean step length significantly shorter when comparing the PCA and tAD groups with controls (*p* = 0.001 or less), but no significant differences were observed when comparing PCA and tAD. When making comparisons between routes for each participant group, step length when walking along the straight corridor was significantly longer than step length walking along the other corridors for all three groups (*p* = 0.013 or less), but differences in step times were small and predominantly did not show any statistically significant differences (see Table 1).

### 3.2 Paths of routes during walking

**Fig. 4:**
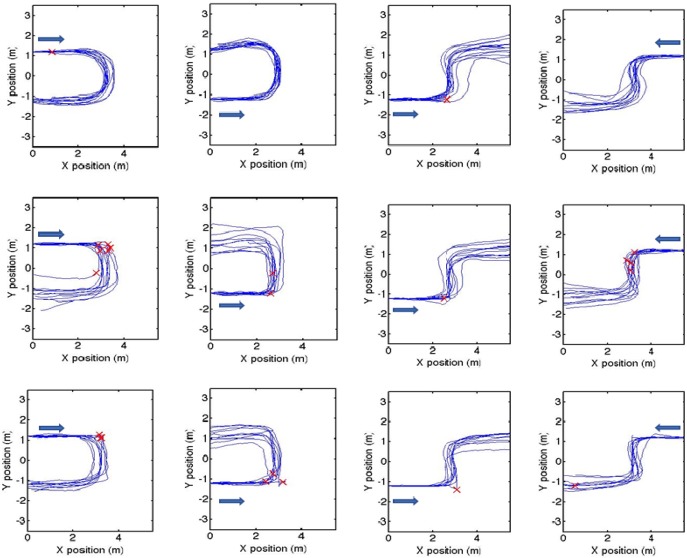
Calculated trajectories for the right foot around the U-shaped (left two columns) and S-shaped (right two columns) corridors, for control (top row), PCA (middle row) and tAD (bottom row), walking in one direction and then returning in the opposite direction (the arrow indicates the direction of walk). The origin was set as the same for all participants, though in practice this was not necessarily the case. Crosses indicate the positions of hesitant steps, defined as a step time >3 standardised residuals from the mean for each participant

Paths for all participants in the three groups are shown in Fig. 4, for walking in both the U- and S-shaped corridors. Observed qualitative differences between paths include sharper right-angled turns for patient groups compared to controls, seen particularly at the first corner. This type of behaviour is illustrated in two attached videos, showing animations of the walking paths for one control participant and one person with PCA, walking along the U-shaped corridor.

### 3.3 Hesitant steps

In Fig. 4, the positions of step times identified as outliers are shown. Clearly, there are some large step times of >2 s for the U-shaped corridor between 2 and 4 m of the individual patient trajectories. 8/139 step times in this region were outliers for the PCA group, and 6/108 step times were outliers for the tAD group. For the S-shaped corridor, 3/111 step times were outliers for the PCA group in the region from 4 to 6 m; for the tAD group there were no outlying step times in this section. For the I-shaped corridor there were very few outliers except for controls where 7/62 step times in 0–2 m region were outliers, all of which occurred very early in the route.

## 4 Discussion

Rigorous investigation of accessibility and mobility issues in people with physical and/or cognitive impairment should ideally be performed in environments that replicate the ‘real-world’ situations that people experience during activities of daily living. Such environments are difficult to set up in conventional motion capture laboratories using opto-electronic motion capture equipment. In this study, we have been able to create a simple scenario involving participants with different presentations of dementia navigating routes. Findings suggest that IMUs can be used in such environments to provide information on clinically relevant behaviour.

Gait was similar in both PCA and tAD groups. Even excluding outliers, participants with dementia had shorter step lengths and longer step times compared with controls. Differences in step times and length were observed when comparing different routes, in agreement with previous observations for adaptive gait in people with AD [22]. Although measures of gait variability in AD are frequently reported in the literature, the overall lengths of the paths walked by the participants were rather shorter than advised for a statistical description of the distribution of step time and step length data [23]. However, the aim of this study was to understand how deviations in step time and length related to specific locations within the tasks. As such, it was far more useful to identify outlier steps, which could then be considered indicators of hesitation. The criterion for defining an outlier step for an individual was based on that individual's step data, rather than using one fixed absolute value of step time as a threshold. In this way, disproportionately increased step times could be reliably identified and located for each individual.

The data show a consistent pattern of hesitation, determined by step time, for some dementia patients during the tasks. The clustering of hesitant steps at particular locations indicates that the approach of outlier detection may be more informative, as compared to a simple description of gait variability. Although it was possible to identify the location of hesitant steps, it is still uncertain what specific characteristics of that location induced this behaviour. The assessment of visually salient features within the visual field at these locations, combined with investigation of eye fixations could further identify problematic environmental characteristics, leading to the potential for design modifications in the built environment to improve mobility.

Tracking the position of the participants used the principle of dead reckoning, i.e. estimation of position from knowledge of initial position and subsequent movement from that position. However, during testing it was difficult to position some participants on a precisely defined mark at the start of each trial, and so this was not included in the protocol. The starting position varied within a square of ∼60 cm, and this position was not recorded. The plots in Fig. 4 show all participants starting from the same point (0,0). This difference between actual and ideal starting position explains some of the variation in the final positions for the complex corridors tasks. All paths are within the range of permissible paths allowed by the physical constraints of the walls. Although effects of sensor drift on walking paths are possible, we have taken steps to mitigate this using zero velocity update. A guide to the amount of drift in *x*- and *y*-positions can be obtained by assessment of drift in the vertical (*z*) direction, which was typically of the order of 5 cm when comparing the start and end of each trial.

Some of the people with dementia took abrupt turns at the corners. The primary importance of visuomotor control in anticipatory actions for adaptive walking is recognised [24, 25]. Controls were clearly able to anticipate the corners and walked with an efficient smooth path around the corners, but some participants in the dementia groups did not demonstrate the same anticipation. Either they were not able to efficiently interpret or act on the visual information, due to low visual orientation or visuomotor/visual processing impairments, or were unable to predict layouts of routes due to diminished topographical processing, spatial memory and/or executive function [26]. Future investigations might explore the relationship between gait dynamics, spatial memory and route learning abilities. Multi-target stepping tests have shown that young people fixate about three steps ahead, but older adults at risk of falling fixate around the immediate target [27].

It was not possible to use conventional techniques for tracking participants and monitoring gait, because the walls blocked any line-of-sight observation of participants. Overall, a total of 2395 steps were processed. In order to make data processing feasible for such a number of steps, a batch processing approach was used, in which a standard threshold of resultant acceleration was used to define swing and stance phases of gait. Although this may impact on the sensitivity and specificity of the processing of step data, the technique has the potential to be used extensively in the assessment of mobility in complex environments, where the portability of the technique and lack of constraints on the physical infrastructure are significant advantages. It is therefore particularly beneficial to be able to use standardised algorithms to process step data. In the context of research into dementia friendly environments, the effects of perceptual environmental variables, such as lighting, visual cues and clutter on patient navigation can be evaluated in a variety of scenarios [26].

The advantage of using the current sensor-based system to track position and measure gait is that no specific infrastructure is required to perform the measurements. The sensors are portable, and no modification of the local environment is necessary. Within the context of AD research, participant spatio-temporal data collection may be facilitated in environments such as day centre or residential care settings. More generally, the technique is applicable to study mobility in the built environment for people with both physical and cognitive impairment. In summary, this work demonstrates the feasibility of IMUs to be used to perform pedestrian navigation and analysis of spatio-temporal characteristics of gait simultaneously. Both patient groups differed from controls but PCA and tAD patients shared similar spatio-temporal gait characteristics. Identification of outlier step times from standardised residuals allowed the identification of locations where participants tended to hesitate.

## 5 Conclusion

It was feasible to use IMU sensor technology to assess how people with AD responded to environmental factors (route complexity) when navigating simulated indoor environments. We consider that such an approach is generally applicable to the investigation of mobility in the built environment.
